# Promoting advanced medical services in the framework of 3PM—a proof-of-concept by the “Centro” Region of Portugal

**DOI:** 10.1007/s13167-024-00353-9

**Published:** 2024-02-22

**Authors:** Fernando J. Regateiro, Henriqueta Silva, Manuel C. Lemos, Gabriela Moura, Pedro Torres, André Dias Pereira, Luís Dias, Pedro L. Ferreira, Sara Amaral, Manuel A. S. Santos

**Affiliations:** 1 University of Coimbra, Faculty of Medicine – Laboratory of Sequencing and Functional Genomics of UCGenomics and Coimbra Institute for Clinical and Biomedical Research (iCBR) Area of Environment, Genetics and Oncobiology (CIMAGO), and Centre for Innovative Biomedicine and Biotechnology (CIBB), 3000-548 Coimbra, Portugal; 2 CICS-UBI, Health Sciences Research Centre, University of Beira Interior, 6200-506 Covilhã, Portugal; 3 Genome Medicine Laboratory, Institute for Biomedicine (iBiMED) & Department of Medical Sciences (DCM), University of Aveiro, 3810-193 Aveiro, Portugal; 4 University of Coimbra, Centre for Business and Economics Research, Faculty of Economics, Av. Dias da Silva, 165, 3004-512 Coimbra, Portugal; 5 University of Coimbra, Centre for Biomedical Law, Faculty of Law, Pátio da Universidade, 3004-545 Coimbra, Portugal; 6 University of Coimbra, Centre for Health Studies and Research and Faculty of Economics, Av. Dias da Silva 185, 3004-512 Coimbra, Portugal; 7 University of Coimbra, Centre for Neuroscience and Cell Biology (CNC) and Centre for Innovative Biomedicine and Biotechnology (CIBB), Rua Larga, 3004-504 Coimbra, Portugal; 8 University of Coimbra, Multidisciplinary Institute of Ageing, MIA-Portugal, Faculty of Medicine, Rua Larga, 3004-504 Coimbra, Portugal

**Keywords:** Predictive preventive personalized medicine (PPPM / 3PM), Personalized medicine, Health literacy, Genomics literacy, Health education, Entrepreneurship, Pharmacogenetics, Therapeutic tailoring

## Abstract

Multidisciplinary team from three universities based in the “Centro” Region of Portugal developed diverse approaches as parts of a project dedicated to enhancing and expanding Predictive, Preventive, and Personalized Medicine (3PM) in the Region. In a sense, outcomes acted as a proof-of-concept, in that they demonstrated the feasibility, but also the relevance of the approaches. The accomplishments comprise defining a new regional strategy for implementing 3PM within the Region, training of human resources in genomic sequencing, and generating good practices handbooks dedicated to diagnostic testing via next-generation sequencing, to legal and ethical concerns, and to knowledge transfer and entrepreneurship, aimed at increasing literacy on 3PM approaches. Further approaches also included support for entrepreneurship development and start-ups, and diverse and relevant initiatives aimed at increasing literacy relevant to 3PM. Efforts to enhance literacy encompassed citizens across the board, from patients and high school students to health professionals and health students. This focus on empowerment through literacy involved a variety of initiatives, including the creation of an illustrated book on genomics and the production of two theater plays centered on genetics. Additionally, authors stressed that genomic tools are relevant, but they are not the only resources 3PM is based on. Thus, they defend that other initiatives intended to enable citizens to take 3PM should include multi-omics and, having in mind the socio-economic burden of chronic diseases, suboptimal health status approaches in the 3PM framework should also be considered, in order to anticipate medical intervention in the subclinical phase.

**Supplementary Information:**

The online version contains supplementary material available at 10.1007/s13167-024-00353-9.

## Introduction

### 3PM concepts go significantly beyond the state-of-the-art in personalized medicine

Despite that nowadays the four nucleotide bases adenine (A), thymine (T), cytosine (C), and guanine (G) have, according to other scientists, replaced the four humors of Hippocrates (blood, phlegm, yellow bile, and black bile) [1], we find important to stress the original context of Hippocrates in the sense of the balance within the body among all “humours” and any particular dis-balance as a cause for specific disease. This standpoint philosophically brings us to the predictive, preventive, and personalized medicine which also emphasizes the balance and complex point of view but with much deeper insights into the molecular mechanisms, including genetics, and interactions with the external factors.

Individual genomic analysis in medical decision-making facilitates the transition towards personalized medicine, leading to novel targeted therapies and a transformative effect on personal health, health economics, and productivity of countries or regions [2]. This type of medicine results from a combination of advancements in genome sequencing, information, and communication technologies [3].

**Fig. 1:**
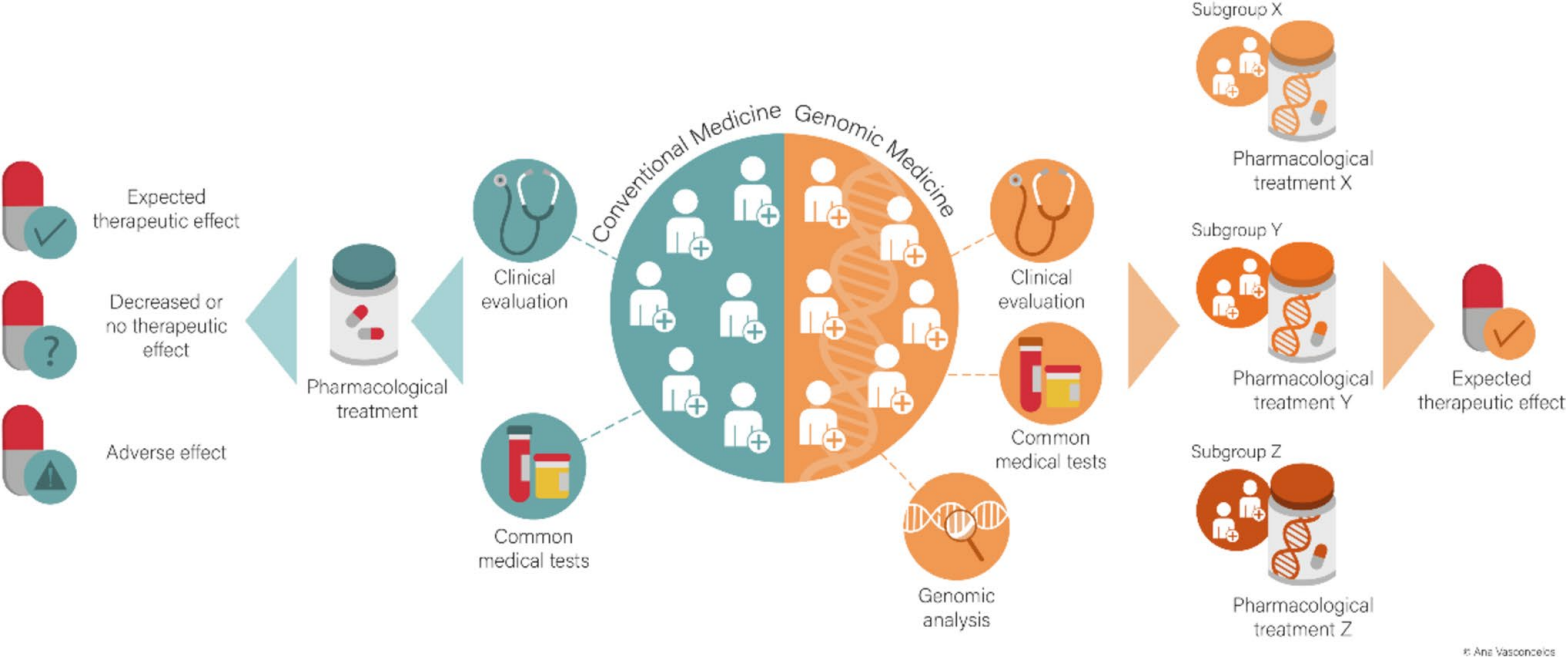
Comparing a treatment by conventional medicine with a treatment in the framework of 3PM utilizing genomic tools. For conventional medicine, patients with the same symptoms should share a common etiopathogenesis and receive similar treatment. The adopted treatment is the one (“one size fits all”) that shows the best results following clinical trials in which various therapeutic approaches are tested. With treatments in the framework of 3PM utilizing genomic tools, patients with the same symptoms are previously subjected to genomic testing and, based on the results, stratified into subgroups (*X*, *Y*, and *Z*) and subjected to treatment algorithms (*X*, *Y*, and *Z*) tailored to individualized patient profiles, in order to achieve the common expected therapeutic effect

Following the European Council Conclusion on Personalized Medicine for Patients [4], “there is no commonly agreed definition of the term Personalized Medicine.” However, it is widely understood that personalized medicine (Fig. 1) refers to “a medical model using characterization of individuals’ phenotype and genotype (e.g., molecular profiling, medical imaging, and lifestyle data) for tailoring the right therapeutic strategy for the right person at the right time and/or to determine predisposition to a disease and/or to deliver timely and targeted prevention” [4].

Personalized medicine should be seen as a patient-centered evolution of medicine and healthcare systems, based on new genetic technologies, to better responding to patient needs [4, 5].

Overall, the main goal of personalized medicine aims at maximizing health benefits, minimizing risks, and gaining in efficiency, the opposite of many conventional medical practices still based on “average patient” or “average doses” concepts. Defining risk groups using genomic markers, personalized medicine allows establishing who should be targeted for early intervention and supports appropriate access to innovative diagnostic methods and better targeted treatment [6].

### Tools and measures instrumental for the paradigm shift from reactive medical services to predictive, preventive, and personalized approaches

Going further, a new paradigm has emerged based on the shift from reactive to proactive medical services in the context of 3PM. 3PM represents a progressing from “disease care” to “health care,” as a new philosophy to tackle the pandemic of common chronic diseases, and the need to encourage macroeconomic, more efficient predictive and targeted preventive measures [7]. 3PM is a new integrative concept in healthcare, which makes it possible to predict individual predispositions before the onset of disease, to provide targeted preventive measures and to create personalized treatment algorithms tailored to the individual [8]. 3PM is effectively promoted by the European Association for Predictive, Preventive, and Personalized Medicine (EPMA) [7, 8].

Areas where we expect 3PM to have a growing impact in the “Centro” Region include cancer, diabetes, neurodegenerative, metabolic, respiratory, cardiovascular, and infectious diseases, but also pharmacogenetics, aging, microbiome, pre-symptomatic carrier identification, reproductive, and fetal medicine [8–10].

The increase in longevity, the increasing prevalence of long-term and complex diseases, and the rise of innovative technologies and scientific breakthroughs and their translation from bench to bedside are putting pressure on the sustainability of health systems in developed countries due to the need to allocate additional resources to health budgets [11]. Health and healthcare sustainability should not be pursued in isolation, but should be addressed for financial, economic, political, legal, ethical, educational, and social reasons [12]. 3PM is emerging as a promise to reverse the ever-increasing costs of healthcare and government health spending in recent years, by harnessing developments in genomics to improve prevention and early diagnosis of disease [6].

Healthcare principles are shifting the focus from illness to health, from treatment to prevention and early diagnosis, from a “one size fits all” approach to personalized medicine [8]. The successful implementation of a 3PM program depends mainly on effective communication with professionals who are able to apply these specific approaches in clinical practice, and with policy makers and administrators who are in a position to make decisions within 3PM, as a prerequisite for effective implementation of 3PM in the health sector [8].

### A proof-of-concept model by the “Centro” Region in Portugal

The “Centro” Region in Portugal corresponds to the central part of its continental territory. “Centro” covers a population of approximately 2.3 million inhabitants, being one of the country’s five mainland regions (NUTS level 2) of direct administration of the Portuguese State. The “Centro” Region is supported by ten main hospitals, having health as one of the priority thematic differentiating domains of research and innovation strategy for smart specialization (RIS3). It is already recognized as an innovation leader in health. Regional strategic goals include health and well-being and biotechnology, two privileged areas for the application and promotion of 3PM.

The main goal of our work was to respond to the main health interests of a citizen today, when concepts and medical applications of genomics are brought into play in the context of 3PM [7]: to receive personalized care that places him/her at the center of health procedures, taking into account his/her potential or actual disease and his/her genetic and life path uniqueness; to receive a medicine that is tailored to his/her allelic variants and is able to provide the right therapy, in the right dose, at the right time, for the right period of time; to predict and prevent possible diseases; and to have access to a medical practice that explains to him/her the basics of understanding the medical procedures that will be applied and their potential ability to restore health.

### Role of EU in supporting new strategies in the framework of 3PM

The activities were developed taking into account the following conclusions of European Union Council on personalized medicine for patients [4], when the Council invites member states to:

- Develop patient-centered policies through patient empowerment and integration of patient perspectives in the development of regulatory processes, in collaboration with patient organizations and other relevant stakeholders
- Integrate advances in human genomics into public health research, policies, and programs
- Develop or strengthen public health communication strategies to improve health literacy and public awareness of existing treatment options, of the benefits and risks of personalized medicine, and of the role and rights of citizens
- Provide education, training, and continuing professional development to equip them with the necessary knowledge, skills, and competences to make the most of the benefits of personalized medicine for patients and health systems
- Promote cross-disciplinary interaction to ensure better understanding of available data, more efficient integration and interpretation of information from different sources, and appropriate decision-making on treatment options
- Consider developing long-term, patient-centered, strategic approaches to address the challenges of access to personalized medicine from a public health perspective, while ensuring the sustainability of national health systems based on patient safety and quality of care

Furthermore, the work carried out fit well with the perspectives addressed by ICPerMed [2]: informed, empowered, engaged, and responsible citizens and healthcare providers; health systems that enable personalized and optimized health promotion, prevention, diagnosis, and treatment for the benefit of citizens and patients; availability and optimal use of health-related information for optimized treatment, care, prevention, and research; and economic value through the creation of next-generation medicine.

### Working hypothesis in the framework of 3PM

Working hypothesis was designed having in mind that 3PM corresponds to a new paradigm based on the shift from reactive to proactive medical services. To support and fostering 3PM, the next few years will also see a paradigm shift in education to health and healthcare, and health literacy, encompassing citizens in general, but also patients, health professionals, healthcare providers, healthcare managers, and policy makers [1]. Thus, working hypothesis is aimed at enhancing and extending the practice of 3PM in the “Centro” Region, elected training of health professionals and capacitation building of citizens in general for a rational use of 3PM, with health literacy as an essential pillar. In addition, a strategic plan was drawn up, and good practices handbooks were written and published to strengthen technical, ethical and legal skills, and entrepreneurship.

### Project design

In order to design a project that would meet the previously identified needs and others listed below (Table 1), a multidisciplinary coordination team and several working teams from different fields of knowledge were set up, coming from the three public universities located in the “Centro” Region of Portugal.

**Table 1:** Activities developed in the framework of 3PM utilizing genomic tools. Activities were designed to build capacity for advanced medical services and support tailoring algorithms aimed at adding value in the framework of 3PM. In order to achieve these goals, activities 1, 2, and 3 include knowledge of the existing offer, planning the response, fostering well-prepared human resources, and investing in the production of handbooks that facilitate and standardize training and promote entrepreneurship. Through activity 4, a strong focus was placed on people, namely, by promoting literacy

**Activity 1: Regional mapping and strategy**
Assessment of the current status of 3PM
Definition of a regional strategy to strengthen the implementation of 3PM
**Activity 2: Training in genomic studies and writing good practices handbooks**
Training human resources in genomic studies applied to cancer, respiratory diseases, and diabetes
Writing good practices handbooks on genomic studies and on ethical and legal issues of genetic information Biolaw, ethics, and biobanks in the framework of 3PM
**Activity 3: Transfer of knowledge and entrepreneurship**
Integration of genomics into the regional network of health units
Support to development of start-ups Writing a good practices handbook on transfer of knowledge and entrepreneurship
**Activity 4: Knowledge dissemination and increasing literacy**
Increasing literacy of health professionals and health students
Increasing literacy of high school students
Increasing literacy of the general population

By involving the three public universities of the “Centro” and their capacitation building on personalized medicine, the project is also aimed at responding to the great challenge of territorial cohesion, mainly when it encourages the dissemination of knowledge and technology concerning innovation on genomics, and the promotion of highly qualified human resources. In addition, the project included partnerships with the main hospitals of the Region, the Regional Directorate of Education, the Portuguese Association of Biology and Geology Teachers, and the Portuguese Press Association.

Some major challenges were considered relevant to be addressed in the “Centro” Region of Portugal (Table 1), namely, appropriate strategies for the implementation of 3PM in the Region; trained people in genomics; availability and dissemination of good practice on genomic studies, legal and ethical concerns, and knowledge transfer and entrepreneurship; integration of 3PM in health units; and raising literacy on the benefits and current limitations of 3PM, among health professionals, students, and the general population.

## Results and data interpretation

### Assessment of the current status of personalized medicine in the “Centro” Region

Comprehensive insights were gained into the current status of 3PM in the “Centro” Region, covering the following key aspects: human and technological resources, research and clinical activities, genome-scale sequencing capabilities, and academic programs offered by universities in the fields of human genetics, bioinformatics, statistics, and advanced computing.

By identifying the resources, competencies, and needs related to 3PM within the “Centro,” a holistic perspective has been developed, which has made it possible to formulate a regional strategy in line with the objectives and aspirations of the Region.

### Definition of a regional strategy to strengthen the implementation of 3PM

Administrators responsible for implementing a 3PM program must bear in mind that this process requires high-quality and real-time information on four critical points: health needs of society, financial resources, operation and infrastructure, and clinical outcomes [13].

One of the outputs from this work was a detailed 240-page document presenting a strategy for the “Centro,” articulated with the National and EU strategies for 3PM. This document was developed through reviewing the literature, from scientific articles to policy documents and other strategic plans, listening to diverse stakeholders in focus groups, performing an inventory of existing capacities, and using the tools of strategic analysis (PESTEL, SWOT, idea generation, and selection). The process and the strategy defined for the “Centro” can guide future efforts to develop strategies for 3PM in other regions.

The following points were considered strategic objectives for the definition of a regional strategy aimed at implementing 3PM: improve the population’s health, reinforce citizens’ participation in their own healthcare, promote social cohesion, protect personal information, ensure financial sustainability, promote value creation, and differentiate the “Centro” Region.

A major distinction was considered between capacitation for genome sequencing, focusing on equipment, and capacitation for interpretation of genomic data, focusing on qualified human resources. Interpretation of genomic data was considered the core competence to be developed in the “Centro.” In addition, the potential for innovation in developing vaccines and treatments based on genome research was highlighted, building on some promising examples in the Region.

Moreover, considering the size of the “Centro” Region in the context of the country and the world, much of the discussion was centered on identifying specific areas in which the Region can be the most relevant. The areas of oncology, neurodegenerative diseases, diabetes, cardiovascular diseases, and eye diseases were identified as very relevant, considering both the existing potential and the population’s needs. Some rare diseases were also identified as a niche to be exploited, based on successful examples in the Region.

Strategic axes and strategic initiatives (Table 2) have been organized in terms of networks, data, equipment and infrastructures, professionals, citizens, and research.

**Table 2:** Strategic axes and objectives for 3PM development in the “Centro” Region. This table summarizes the formulated strategy for 3PM development in the “Centro” Region, presenting the axes that will guide strategy implementation and the main objectives in each of them

Strategic axes	Strategic objectives
Networks	Promote the cooperation, the exchange of knowledge and joint projects related to 3PM Integrate genetic and clinical data Institutionalize and promote the networks in the “Centro” Region Ensure the comparability between outputs produced by different laboratories in the Region
Data	Contribute to the protection and privacy of data Promote consensus on data sharing and protection
Equipment and infrastructure	Ensure shared access to equipment and technology to implement and develop 3PM in the Region Manage the sequencing capacity in the “Centro” Region
Professionals	Empower health professionals for 3PM Retain talent in the Region
Citizens	Promote citizens’ perception of key 3PM concepts Reinforce the citizens’ participation
Research	Promote translational research Increase funding for research projects related to 3PM Stimulate innovation and entrepreneurship

Implementing such a strategy will require resources. Thus, policy makers must be aware of their ability to pay, having in mind distributive justice and proportionality, to accomplish accessibility, affordability, and system readiness [13]. More generally, decision makers and health stakeholders wish to know if economic value is provided. Although the economic value of 3PM is still difficult to quantify, a few studies already indicate advantages, compared to more traditional approaches in medicine [14–16]. By reducing the waste and costs associated with ineffective treatments and their side effects, 3PM also promotes the rational and efficient use of available resources and has the potential to relieve pressure on national health services [17]. For this reason, the strategy document devoted a chapter to provide an overview of existing evidence, anticipating the benefits that could be expected.

A systematic review found robust evidence for the cost-effectiveness of genotyping before treatment with several common drugs [18]. In the majority of studies, the increased costs of genotyping were more than offset by the positive effects on patients’ life expectancy and quality of life [18], with the reduction of the risk of adverse drug reactions being a key benefit [19]. Nevertheless, some studies (e.g., [19, 20]) show that the cost-effectiveness of implementing pharmacogenomics-guided drug use varies according to the diseases and types of drugs evaluated, which supports our choice of highlighting specific areas in the strategy document. Other studies mentioned cost-effectiveness evidence of using targeted massive parallel sequencing (MPS) as routine screening for allelic variants associated with maturity onset diabetes of the young [21] and the cost-effectiveness of an anticipatory intervention for asthmatics based on a relevant genetic abnormality [22]. Moreover, it is also noted that 3PM is an emerging paradigm in cancer treatment, aiming to tailor cancer therapy to the individual patient and treatment scenario, although cost-effectiveness analyses face several challenges and remain unclear [23].

### Training personnel in genomics

Sequencing parts of the genome, the exome or all the whole of an individual genome, is becoming an affordable method to attain significant knowledge regarding rare and common genetic variations, especially those that are functionally significant [24]. Therefore, being proficient in sequencing is crucial to establish expertise in genomics. The training was developed using the laboratory from the university with specific expertise, namely, in cancer, respiratory diseases, and hereditary forms of diabetes.

Deep phenotyping plays a crucial role in cohort stratification for population-based studies, like genome-wide association studies (GWAS) and polygenic risk scores (PRS). This approach significantly improves the predictability of models that solely rely on genetic information.

Training in genomic studies is aimed at ensuring the acquisition of technical skills. Effective training is essential in diagnosis, therapy, and research areas related to 3PM to enable customization of treatment algorithms according to individual patient profiles. Patients with allelic variants that are directly targetable may gain benefits from proven or novel therapies which could enhance their survival and quality of life. Main application fields are stratification of cancer situations, characterization of genetic diseases and pharmacogenomics. By reducing the time required for diagnosis and increasing the accuracy of identifying the molecular mechanism, genomic research facilitates prescribing the most suitable treatment, while also enhancing cost-effectiveness of patient treatments. Additionally, genomic research can assist physicians in predicting a patient's functional status prior to disease onset, or before irreversible conditions emerge, and thus promoting and improving the health of the population. This enhances 3PM by enabling the prioritization of preventative measures and methods of treatment [25].

### Good practices handbook on genomic studies

The writing of a good practices handbook on genomic studies was based on the following assumptions [26–28]:

- Next-generation sequencing (NGS) has revolutionized our ability to decode genome-scale DNA information, ushering rapid advancements in both fundamental research and clinical applications
- The adoption of NGS has compelled laboratories to embrace greater complexity in their workflows, accompanied by an increased demand for sophisticated bioinformatics analysis
- Although ongoing advances in technology and equipment have gradually reduced the cost of NGS, it still remains a relatively expensive technology, limiting its accessibility to a few laboratories
- The effectiveness of NGS protocols hinges heavily on precise sample preparation and meticulous data analysis. To optimize cost-efficiency and time savings, it is imperative to provide comprehensive training to all professionals involved and adhere to best practices, techniques, and methodologies

This handbook was entitled “Sequenciação de DNA—NGS” and published as an open access publication in Portuguese [29].

### Good practices handbook on ethical and legal issues of genetic information

A good practice handbook was conceived as a guide dedicated to ethical and legal concerns generated by the rapid advances of health and healthcare in the framework of 3PM utilizing genomic tools. High quality of ethical standards is a prerequisite for successful implementation of 3PM in healthcare systems [7]. The handbook, based on national and international legislation, produced ethical thinking aimed at health professionals. As many of the challenges arising from this new paradigm of medicine and healthcare provision do not find neither sufficiently clear answers at the regulatory level, nor established ethical thinking, the handbook also presents new thinking and proposals on ethical and legal challenges generated by 3PM.

The handbook addresses issues in the fields of biobanks and DNA profile databases; conflicting interests between family members regarding access to genetic information and the duty of secrecy; automated decision systems; genomic-based digital health and medicine; impacts of personalized medicine on public health, social justice, and personal discrimination on the grounds of genetic heritage; the role of EU as a driver of personalized medicine; and implications of personalized medicine in health professions. The handbook was entitled “Medicina Personalizada de Base Genómica, Boas Práticas, Ética e Direito” and published as an open access publication in Portuguese [30].

### Biolaw, ethics, and biobanks in the framework of 3PM utilizing genomic tools

Using individual’s genomic information in disease prevention, diagnosis, and personalized treatment requires physicians to know more than simple genetic bases to develop genetic rationales underlying an adequate selection of genetic or genomic testing, interpretation of test results, and clinical decision. That implies specific education, skills and experience, and mastery of the “state of the art,” this is understood as the generally accepted clinical practice.

Using genomic information has particular implications for healthcare professions [31]. For this reason, medical genetics services should be installed in hospitals and healthcare institutions that must be aware of the increased responsibility of physicians dealing with genetic information, namely, in the field of genetic counseling. Adequate training of health professionals must be ensured.

Relevant aspects concerning the process of introducing genomic studies in the field of public health should be considered [32–34]. The process must be progressive and cautious, always ensuring that all decisions are taken based on scientific evidence and minimizing any threats and risks to the human and fundamental rights of citizens. It is necessary to promote health and genomic literacy, to empower citizens to make truly informed decisions regarding their health status, with clear advantages in terms of public health. Despite the aspiration for the broadest possible introduction of 3PM into the National Health Service, special care is needed to ensure equitable and non-discriminatory access for all citizens.

In 3PM, the physician–patient relationship is often solely guided by obtaining and knowing the diagnosis, with the transmission of genetic information about diagnosis playing the main role. Additionally, ethical concerns are overwhelming.

Physicians failing to follow the “state of the art” can fall under legal liability and be sued due to negligence or personal injury. However, evidence based on genetic/genomic information is, frequently, insufficient to support the definition of a general accepted clinical practice or a standard to provide physicians guidance [31].

In order to avoid any liability for possible diagnostic errors, two issues are paramount. One is the provision of reinforced informational duties, which are the basis for obtaining duly informed consent, especially regarding the lack of immediate therapeutic benefit. Another one is to comply with the “state of the art” when making a diagnosis, considering any and all obvious symptoms and recording a well-made and detailed analysis of all tests, procedures, and resources on available devices and instruments [35, 36].

Besides the production of a good practices handbook on ethical and legal concerns of genetic information, the biolaw team was still involved in the preparation of proposals for legislative changes at national and European level, in the development of adequate procedures to obtaining informed consents; in the analysis of ethical, legal, and social aspects of personalized medicine; and in initiatives dedicated to health literacy promotion and to empowering patients organizations.

In the field of biobanks [37], the biolaw team defined the legal regime that establishes the conditions for treatment of biological samples of human origin (from collection, processing, analysis, availability and use, storage, and destruction) for the purposes of fundamental, applied, or translational scientific research; established that a financing guarantee should exist to avoid compromising the essential ethical and scientific exemption of structures dedicated to biobanking activities for scientific research purposes; and established that technical measures should ensure the safety and quality of procedures, by monitoring compliance with them.

Concerning automated decision systems in 3PM, the biolaw team identified as essential [38, 39]: to pay attention to basic principles of General Data Protection Regulation; the indispensability of subjective analysis of patient’s condition and human supervision, action, and recommendation of the predictions achieved by automated systems; and an urgent social debate, mainly involving people working within the health system.

## Transfer of knowledge and entrepreneurship

Unlike the development of new drugs, whose development, patenting, and licensing processes have extremely high costs, even prohibitive for small countries, the costs of innovation in 3PM are affordable, as long as new models of health management and administration are implemented and take into account the specificities of incorporating predictive, preventive, and personalized approaches in medical decisions.

With this project, the “Centro” is trying to lead the way in 3PM and build the conditions to tackle the shortcomings generated by models that are not suited to the implementation and development of 3PM. One of the pursued conditions consists of drawing up a specific strategic plan. Another is to develop conditions that allow the Region to benefit from the high potential of 3PM for producing patents and start-ups in the areas of diagnostic technologies, digital medicine, and artificial intelligence, as well as in the fundamental and sensitive area of managing individual genomic data.

Promoting start-ups creation will encourage entrepreneurship, transfer and enhanced knowledge, support management of intellectual property, and support seed phase of start-ups and procedures to fundraising.

To foster entrepreneurship and the creation of start-ups, several actions were considered in the project and put in place: promote 3PM through seminars, workshops, and visits to successful case models; support academic researchers in the field of 3PM to translate their research into industry and health services; provide training in intellectual property directed to those involved in research and development in the field of 3PM; offer mentorship in the early stages of development of start-ups and spin-offs in the field of 3PM; and identify funding opportunities in the field of 3PM and provide support in the submission of proposal applications. To facilitate the dissemination of the procedures underlying these approaches, a good practices handbook has been written.

### Good practices handbook on transfer of knowledge and entrepreneurship

This handbook aims at guiding researchers and health professionals in 3PM, promoting knowledge transfer and entrepreneurship in the field. Genomics research progresses rapidly, providing great opportunities to translate discoveries into industry and healthcare. However, researchers do not always realize the essential steps to generate economic value from their findings. This guide aims to raise awareness about entrepreneurship and intellectual property and provide guidance to researchers in the initial stages of creating start-ups and spin-offs in the field of genomics-based 3PM. It is hoped that this guide will help to bridge the gap between research and industry in 3PM.

## Knowledge dissemination and increasing literacy

### Capacity building through increasing literacy of health professionals and health students, high school students, and general population

In general, health professionals face real difficulties to integrate scientific and technological genomic advances in standards of medical practice, to communicate with consultants when dealing with them, and to face associated legal and ethical concerns. And what about common laymen, when it is expected they are able to assume the right to autonomy and to make choices and sign informed consents, all that implying to know and understand a minimum about genetics and genomics?

These difficulties were addressed through dissemination initiatives directed to health professionals and health students: webinars for academic community and health professionals, proposal of internships for health students in sequencing laboratories of the universities involved in the Project, and a “Genomic Medicine Day” to bring together health professionals (physicians, researchers, and nurses) and motivate thinking and discussion on genomic aspects. Webinars included topics on genomics and its applications in medicine, ethical and legal dimensions, genomic databases, genomics in diagnostics and therapeutics, and health economics.

An approach to education for genomics, aimed at pre-university schools, justified the achievement of webinars for secondary school teachers of Biology, including topics on genomics and their applications in medicine, ethics, and biomedical law; proposal of summer internships for secondary school students and selective visits to genomics laboratories; multimedia production for high school students and teachers concerning concepts and perspectives in the field of genomics; production of an illustrated book entitled “Genomic Medicine”; and the “Genomic Medicine Day,” also for students and teachers from diverse levels of pre-university education.

Education aimed at general population was based on publication of texts related to personalized medicine and genomics in newspapers of the Region, publication of a cartoon in a national newspaper to raise awareness of the community, the writing and publishing of the illustrated book “Genomic Medicine,” writing and performing of two theater plays addressing issues on genomics and personalized medicine, creation of a specific website, and using social networks and press releases to disseminate activities.

### Survey on genomic literacy

A survey was prepared to assess literacy in higher education students. The GKQ (genetic knowledge questionnaire) was selected for this research, through bibliographical research on existing measuring instruments to assess genetic literacy [40]. After the author’s authorization, the questionnaire was translated and culturally adapted into Portuguese. The study was approved by Ethics Committee of the Faculty of Medicine of the University of Coimbra. Pre-test was already applied to students at the University of Coimbra.

### Science communication and general population engagement in 3PM issues

Science communication plays a vital role in fostering an aware and informed society, enabling the demystification and effective dissemination of scientific concepts. Effective and accurate science communication is paramount, to enabling greater health and healthcare literacy and informed decision-making among the general population. Communication strategies and societal engagement should be tailored to diverse target audiences, encompassing various age groups and knowledge domains and utilizing a range of communication and interaction channels.

Issues related to genetics and genomics, advances in research and their applications and limits, and the increasingly personalized nature of medicine are hot topics today, with 3PM issues standing out, as deserving special consideration. A communication strategy centered around 3PM was carefully designed to align with citizen’s related knowledge, interests, and opinions.

Our aim was to employ artistic forms of expression, to convey this theme and stimulate public interest, thus enhancing health literacy. Furthermore, we sought to cultivate critical thinking and elevate public enthusiasm for science, through content dissemination, thereby fostering a more engaged society that is attuned to the profound impacts of the rapid growth of scientific knowledge with invoked or real medical applications. In the context of the project, different forms of artistic expression were used to communicate genomic medicine: an illustrated book and theater performances.

### Illustrated book

**Fig. 2:**
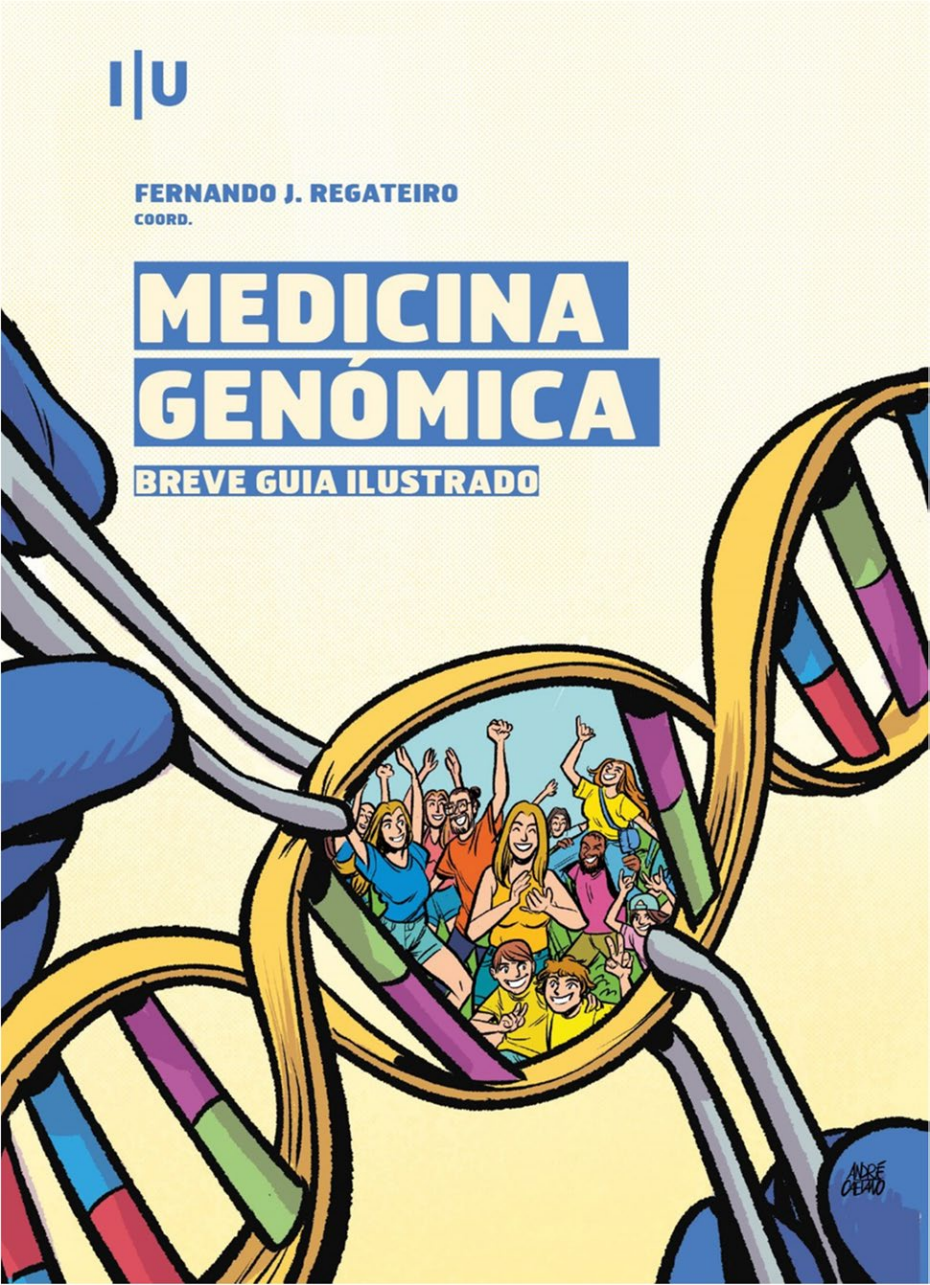
Cover of the book “Medicina Genómica: Um Breve Guia Ilustrado.” This book covers fundamentals, study methods, and applications of genomics. It was written aiming at promoting citizens’ literacy and scientific culture, to support more informed decisions in the framework of 3PM. The use of artistic languages, such as visual storytelling and illustration, has been recognized as a highly valuable tool for conveying scientific concepts in a simple and engaging manner to society. The book was published as an open access publication in Portuguese [42]

The use of artistic languages, such as visual storytelling and illustration, has been recognized as a highly valuable tool for conveying scientific concepts in a simple and engaging manner to society [41]. In pursuit of this objective, a multidisciplinary team was assembled to develop a co-creation strategy for an illustrated book on genomic medicine (Fig. 2). This effort involved organizing meetings with experts from diverse fields of knowledge, including scientific research, medicine, biomedical law, health economics, science communication, and collaborating closely with the illustrator responsible for creating the book’s visuals. The creative process was the result of this collaborative work. A glossary was written, describing the meaning of relevant words or processes to facilitate the reading and understanding of the book. The illustrated book was subsequently edited and published in open access [42].

### Theater

One of the foremost challenges confronting modern science is the development of inventive approaches to engage society with scientific knowledge. In an effort to foster creativity and captivating modes of communication, the utilization of artistic languages to delve into scientific topics has demonstrated immense promise. Theater serves as an illustrative example, as it possesses the ability to evoke emotions and bring attention to diverse concerns. This form of artistic expression, as a result, holds the potential to captivate individuals in particular subjects, including science, along with ethical, social, and political dimensions intertwined with it [43]. Taking advantage of this powerful tool for communicating science, two plays mainly directed to genetics and genomics were produced by students and professionals in artistic studies, entitled “Genetic Manipulations” and “Alice and the Wonders of Genes.” The plays were presented at the University of Coimbra.

Responses to a concise survey distributed to attendees of the theatrical performances suggest the plays contributed to bridging the gap between society and 3PM and indicate that plays had a positive impact on the perception of non-specialist audiences. In alignment with prior research [43], we believe that this experience holds significant potential for enhancing health literacy. Further research should be conducted with a larger and more diverse population sample and encompassing various themes addressed in the plays, to improve the perception of the impact.

## Discussion and open questions

The discussion focuses on accomplishments that surpass the “state of the art,” by producing innovative materials relevant to areas inaccessible to much of the population, employing new communication methods, and adapting uninitiated solutions to 3PM scope. The achievements include the following examples which surpass the “state of the art” and help to:

- Create a consortium for 3PM, by bringing together researchers from varied fields (health, business/economics, law, technology, and communication) from the three public universities of the “Centro” Region
- Devise a regional strategy that uses genomic tools to develop advanced healthcare, ensuring the Region maintains its international competitiveness in the healthcare sector within the framework of 3PM
- Train personnel with highly qualified laboratory skills
- Increase balanced regional distribution of technical knowledge on genomics
- Define and produce recommendations and three good practices handbooks
- Build capacities for personalized medicine through knowledge dissemination among health professionals
- Develop information technology resources
- Support the creation of start-ups
- Define legal and ethical requirements
- Disseminate knowledge and educational initiatives to health students, high school students, and the general public, aimed at promoting the understanding and appropriate use of personalized medicine
- Customize strategies to meet the needs of the specific target audience, addressing concerns expressed by civil society
- Form multidisciplinary teams to develop and implement projects
- Present complex concepts through simple narratives
- Leverage artistic techniques, like theater and illustration, to complement medical terminology and enhance comprehensibility of foundational concepts

The activities and outcomes mentioned earlier align seamlessly with the strategic options outlined for health by the “Centro” Region. As a health innovation leader, it was necessary to develop frameworks that facilitate the widespread use of predictive, preventive, and personalized medicine. The project was an apt and timely tool to achieve this goal. The results are consistent with the project’s ambitious vision.

Considering that the “Centro” Region of Portugal registered the lowest percentage of young people (0–14 years) (11.8%) and, along with the “Alentejo” Region, the highest percentage of elderly population (27.0%) [44], this project is also relevant for this Region, namely, taking in account the preventive dimension of 3PM. Preventive medicine is a cornerstone of health system sustainability and improved quality of life, providing populations with more healthy years of life, namely, through suboptimal health status approaches in the 3PM framework [45]. This approach allows the identification of early phases of diseases, e.g., reversible stage of chronic diseases, even before clinical manifestations take hold.

Investing in projects aimed at developing advanced medical services in the framework of 3PM utilizing genomic tools in health sectors such as cancer, metabolic, and respiratory diseases can also yield a favorable return on investment. This is due to the positive externalities they generate, including the reduction of ineffective treatments and adverse drug reactions at both individual and population levels, the promotion of a more rational and efficient use of resources, an average increase in QALYs, and a relieving of pressure on national health services [17–23].

### Open questions

Bearing in mind that effective and efficient adoption of 3PM as a common resource in health and healthcare demands more than just knowledge of genomic bases, other dimensions must be considered to induce a change in people’s attitudes and knowledge. This includes recognizing citizens as stakeholders and managers of their health condition, not just customers or recipients of healthcare provision, prioritizing the relevance of education in the broadest sense (including family, school, informal belonging spaces and their members, and the internet), addressing legal and ethical concerns, protecting health data, and valuing the role of patient organizations.

Concerning activities aimed at capacity building through health and healthcare literacy, it is important to temper enthusiasm with a clear statement of the methodologies we rely on. When it comes to 3PM that uses genomic tools, cautionary language should be employed:

- Although genetic information has the ability to personalize treatments based on individual expected response to a therapeutic molecule, it is important to consider the expression of genetic information and environmental factors, as these can impact the outcomes. Therefore, achieving a desired outcome necessitates consideration of personal genetic data and its anticipated or confirmed expression, as well as potential interactions among expressed gene products involved in a specific drug’s metabolism and relevant environmental factors
- Furthermore, one must also be aware of the comparative constraints of developing advanced medical services in the framework of 3PM utilizing genomic tools in contrast to multi-omics research, particularly when targeting the identification of genes, RNA, proteins, metabolites, and medical imaging [46]. Multi-omic studies enable the detection of a wider range of biomarkers, which enhances the possibility of identifying new drug targets and predictive, diagnostic, or prognostic indicators for treatment stratification, early detection, or estimation of patient clinical outcome. This is highly relevant to disease prediction and prevention, as demonstrated for tumors [46]

The preliminary survey conducted among secondary school students indicates that the illustrated book on Genomic Medicine has the potential to enhance knowledge in genomic medicine by improving 3PM literacy. After reading this book, there was a noticeable increase in the number of correct responses relative to prior assessments. However, a larger survey is required to formulate more conclusive results.

Regarding theater, responses to a short survey given to attendees of the performances suggest that the plays helped bridge the gap between society and 3PM, and that they had a positive impact on the perception of non-expert audiences. Consistent with previous research [43], we believe that this experience has significant potential for improving health literacy. Further research is necessary with a broader and more diverse population sample and incorporating various themes addressed in the plays to enhance the impact perception.

## Conclusions, expert recommendations, and outlook

### Aims and principles

Aimed at enhancing and expanding the use of 3PM in health and healthcare in the “Centro” Region of Portugal, our approach encompassed strategic planning and training in genomic testing, addressed ethical and legal concerns, developed approaches to support entrepreneurship and start-ups creation, and fostered citizens’ education through conventional and innovative approaches.

“Knowing to make lasting changes” was the fundamental principle we followed, namely, to define the axes and initiatives of a regional strategy to strengthen the implementation of 3PM.

### Capacitation building to 3PM, through education and a cultural evolution

Only educated people—people who value what they use because they know what it means—can use a resource properly and get the most out of it. This is clearly the case when disease prediction, prevention, and early diagnosis are a primary goal. Changing people’s attitudes and behavior to value their own role in the process is the stepping stone to success [47]. For this reason, the project included an important part of the effort to empower citizens to take greater responsibility for their health, when addressing approaches to increase 3PM literacy among health professionals, students, and the general population.

Educated individuals who have an interest in health and healthcare are inclined to participate in health-related matters, as they hold health, prevention, disease prediction, early diagnosis, and tailored treatment in high esteem.

Improved health and health literacy are crucial in achieving the goals of this project, necessitating ongoing education to ready citizens for transformative approaches. Taking genomic testing as an illustration, individuals must understand not only what a genetic test is but also its restrictions, hazards, advantages, and probable uncertainties [24]. This represents a cultural evolution.

Responses of people after attending theatrical performances suggest the plays indicate they had a positive impact in the perception of issues associated with 3PM [43].

Two preliminary surveys indicated that certain approaches to boost literacy, specifically the reading of the book “Genomic Medicine” and watching theatrical productions, were effective. However, further surveys must be conducted and evaluated in these and other areas to bolster conclusions or make necessary adjustments.

### Outcomes on technical training and entrepreneurship

Adding significant value and bring about lasting transformative effects should result from the support aimed at development of start-ups and knowledge transfer. Writing the good practices handbook on transfer of knowledge and entrepreneurship should also contribute to this goal.

In the same vein, we proceed with the training of human resources in genomic technologies and the writing of the good practices handbooks to serve as guidance in next-generation sequencing and address ethics and legal issues.

A practical result has been already recorded as a recent creation of a start-up in the genomic field, associated with work carried out within the scope of our project.

### Serving the shift of paradigm from reactive to 3PM needs continuity

The prevalence of reactive paradigm underlying nowadays organization of healthcare services raises avoidable suffering for patients, makes treatment more difficult or impossible and excessively burdens health budgets. In many ways, instead of a “National Health Service,” we have a “National Sickness Service.”

A paradigm shift from reactive to 3PM should reverse the harmful consequences of the reactive healthcare paradigm mentioned above and improve overall quality of medical services, making them more effective and more efficient and available for any socio-economic group in the society [6–8].

The short duration of the project did not allow for consolidated results in some aspects but rather acted as a proof-of-concept that deserves continuity to make it more effective.

Overcoming illiteracy is a continuous process that requires efforts to educate and inform citizens, enabling them to make conscious and responsible decisions in the demanding and challenging arena of 3PM. This is yet another strong reason to support and promote future development in this field.

The relative novelty of 3PM approaches, the relative lack of personnel trained in genome research and analysis of results, the illiteracy in this area, the fact that the “Centro” Region is recognized as a leader in health innovation, and the successful creation of a large team bringing together researchers from the three public universities in the Region are additional reasons for setting up future projects to deepen and densify some of the results already recorded.

Furthermore, an effective and efficient implementation of 3PM should be a multicenter and multinational effort, requiring the creation of reference centers and the establishment of expanded networks for clinical, scientific, and economic reasons.

### 3PM is much more than sequencing the genome or the exome

Furthermore, we should stress that sequencing parts of the genome, the exome or even the whole genome of an individual, are only a fraction of the technologies that should be implemented in the framework of 3PM. Genomic tools are relevant, but they are by far not the only resources 3PM is based on. 3PM is much more complex—that is a holistic attitude towards healthcare.

New initiatives intended to enable citizens to take 3PM should broaden the areas to be dealt with, namely:

- Multi-omics [46], in order to bolster predictive, preventive, and personalized medicine
- And, having in mind the socio-economic burden of chronic diseases, valuing suboptimal health status approaches in the 3PM framework [45], should be helpful to identifying chronic diseases in a reversible stage, before clinical manifestations take hold

### Supplementary Information

Below is the link to the electronic supplementary material.

Supplementary file1 (DOCX 21 KB)

## Data Availability

Not applicable.

## Abbreviations

3PM: Predictive, Preventive, and Personalized Medicine
DNA: Deoxyribonucleic acid
EU: European Union
GKQ: Genetic knowledge questionnaire
GWAS: Genome-wide association studies
ICPerMed: International Consortium for Personalized Medicine
MPS: Massive parallel sequencing
NGS: Next-generation sequencing
NUTS: Nomenclature of territorial units for statistical purposes
PESTEL: Political, Economic, Social, Technological, Legal, and Environment
PRS: Polygenic risk scores
PPPM: Predictive, Preventive, and Personalized Medicine
QALY: Quality-adjusted life year
RNA: Ribonucleic acid
SWOT: Strengths, weaknesses, opportunities and threats
