# Supplementary material for: Promoting advanced medical services in the framework of 3PM—a proof-of-concept by the “Centro” Region of Portugal
Source: EPMA J. 2024 Feb 22;15(1):135–48. doi: 10.1007/s13167-024-00353-9 (PMC10923757; doi:10.1007/s13167-024-00353-9)
Supplement: Supplementary file 1 — Supplementary file1 (DOCX 21 KB) [file 13167_2024_353_MOESM1_ESM.docx]

**PPPM innovation highlights**

**Manuscript:** Promoting advanced medical services in the framework of 3PM – a proof-of-concept by the "Centro" Region of Portugal **(**Fernando J Regateiro et al.)

1. **Working hypothesis in the framework of PPPM**

Capacitation building for advanced medical services in the framework of 3PM using genomic tools, has been addressed through:

- Regional mapping and strategy: assessment of the current status and definition of a regional strategy to strengthen the implementation of 3PM;
- Training in genomic studies and writing good practices handbooks on genomic studies and on ethical and legal issues of genetic information;
- Transfer of knowledge and entrepreneurship: integration of genomics into the regional network of health units; support to development of start-ups; writing a good practices handbook on transfer of knowledge and entrepreneurship;
- Knowledge dissemination and increasing literacy of health professionals and health students, high school students, and general population.

1. **Innovation towards the predictive approach, targeted prevention and personalisation of medical services**
2. **Predictive approach**

The predictive approach has been achieved by ensuring some of the supports to determine the presence of risk or susceptibility to a genetic disease and the response to treatment:

- Creating conditions, through capacitation, that allow citizens and health professionals to be at the forefront, ready to become social and technical transforming agents;
- Training human resources in advanced genomics, using in particular expertise from genomic studies of cancer, respiratory diseases and metabolic diseases (MODY), which are among the areas defined as strategic by the "Centro" Region;
- Production of two handbooks outlining best practices in genomics and ethical and legal concerns - these publications are currently scarce or non-existent.

1. **Targeted prevention**

A targeted prevention approach has included activities designed to prevent the onset of preventable diseases or to promote early intervention to prevent the full expression of a disease. This implies the ability of people to interpret facts, create knowledge and, through education, to judge what is good and what is bad, and finally to make personal choices, and to commit and act accordingly. This has been achieved:

- With the work to improve literacy, education and critical thinking in the context of 3PM, using genomic tools, as a relevant approach for individuals to take responsibility for their health and adopt lifestyles and behaviours of a preventive nature;
- Developing a communication strategy to increase literacy in genetics and genomics in the context of 3PM, in line with citizens' knowledge, interests and opinions;
- The creation and performance of two plays, "Genetic Manipulations" and "Alice and the Wonders of Genes", by students and professionals coming from artistic fields;
- Designing and writing an illustrated book through a creative process aimed at communicating scientific concepts to society in a clear and engaging way.

1. **Personalization of medical services**

The personalisation of medical services, which aims to develop medical services based on a person's genetic make-up and their disease or susceptibility to disease, has been achieved:

- With the adoption of the principle "first know, then change": to support the design of a new regional strategic plan; to strengthen the implementation of 3PM; and to assess population literacy;
- Involving the main hospitals of the "Centro" Region as partners in the project;
- Raising awareness among health professionals and medical students of the benefits of advanced medical services in the context of 3PM using genomic tools.

1. **How does the presented innovation go beyond the state of the art contributing to the paradigm shift from reactive medicine to PPPM?**

- Defining the basis for a new strategic plan for the "Centro" Region, a leader in health innovation, and the accumulated clinical experience based on genomic tools enable widespread utilization of 3PM in the Region;
- Developing activities aimed at preventive dimension of 3PM, to provide populations with more healthy years of life – “Centro” is the Region of Portugal with the lowest value (11.8%) for young people (0-14 years), and the most significant value of the elderly population (27.0 %);
- Fostering development of advanced medical services in the framework of 3PM, which reduces ineffective treatments and adverse drug reactions, promotes a more rational and efficient use of resources and an average increase in QALYs, and contributes to relieving of pressure on national health services;
- Promoting sustainability of the expansion of 3PM utilizing genomic tools, through a shift of people's attitudes and knowledge based on increasing of specific literacy and education, namely on ongoing education;
- Bridging the gap between society and 3PM through theatre – responses to a short survey given to attendees of the performances suggest that plays had a positive impact on the perception of the issues included in the plays, by non-expert audiences;
- Improving a better comprehension of 3PM, as a prerequisite for its efficacious utilisation, by increasing genomics literacy and empowering individuals to take greater responsibility;
- Considering the aforementioned factors and interventions as facilitators in expediting the shift from reactive healthcare to PPPM.
